# Group B Streptococcus Hijacks the Host Plasminogen System to Promote Brain Endothelial Cell Invasion

**DOI:** 10.1371/journal.pone.0063244

**Published:** 2013-05-02

**Authors:** Vanessa Magalhães, Elva Bonifácio Andrade, Joana Alves, Adilia Ribeiro, Kwang Sik Kim, Margarida Lima, Patrick Trieu-Cuot, Paula Ferreira

**Affiliations:** 1 ICBAS- Instituto de Ciências Biomédicas de Abel Salazar, Universidade do Porto, Porto, Portugal; 2 IBMC- Instituto de Biologia Molecular e Celular, Porto, Portugal; 3 UFP- Universidade Fernando Pessoa, Faculdade de Ciências da Saúde, Porto, Portugal; 4 Department of Pediatrics, The Johns Hopkins University School of Medicine, Baltimore, Maryland, United States of America; 5 Department of Hematology, Hospital de Santo António (HSA), Centro Hospitalar do Porto (CHP), Porto, Portugal; 6 Institut Pasteur, Unité de Biologie des Bactéries Pathogènes à Gram-Positif, CNRS ERL3526, Paris, France; Instituto Butantan, Brazil

## Abstract

Group B Streptococcus (GBS) is the leading cause of meningitis in neonates. We have previously shown that plasminogen, once recruited to the GBS cell surface and converted into plasmin by host-derived activators, leads to an enhancement of bacterial virulence. Here, we investigated whether plasmin(ogen) bound at the GBS surface contributes to blood-brain barrier penetration and invasion of the central nervous system. For that purpose, GBS strain NEM316 preincubated with or without plasminogen plus tissue type plasminogen activator was analyzed for the capacity to adhere to, invade and transmigrate the human brain microvascular endothelial cell (hBMEC) monolayer, and to penetrate the central nervous system using a neonatal mouse model. At earlier times of infection, plasmin(ogen)-treated GBS exhibited a significant increase in adherence to and invasion of hBMECs. Later, injury of hBMECs were observed with plasmin(ogen)-treated GBS that displayed a plasmin-like activity. The same results were obtained when hBMECs were incubated with whole human plasma and infected with untreated GBS. To confirm that the observed effects were due to the recruitment and activation of plasminogen on GBS surface, the bacteria were first incubated with epsilon-aminocaproic acid (εACA), an inhibitor of plasminogen binding, and thereafter with plasmin(ogen). A significant decrease in the hBMECs injury that was correlated with a decrease of the GBS surface proteolytic activity was observed. Furthermore, plasmin(ogen)-treated GBS infected more efficiently the brain of neonatal mice than the untreated bacteria, indicating that plasmin(ogen) bound to GBS surface may facilitate the traversal of the blood-brain barrier. A higher survival rate was observed in offspring born from εACA-treated mothers, compared to untreated mice, and no brain infection was detected in these neonates. Our findings suggest that capture of the host plasmin(ogen) by the GBS surface promotes the crossing of the blood-brain barrier and contributes to the establishment of meningitis.

## Introduction

Group B Streptococcus (GBS), a common designation for *Streptococcus agalactiae*, is the leading cause of neonatal infectious diseases comprising pneumonia, sepsis, and meningitis [1]. Despite antibiotic therapy, the associated mortality remains high (approximately 10%) and up to 50% of surviving infants experience permanent neurological sequelae as deafness, seizures, hydrocephalous, cerebral palsy, and cognitive deficits [2], [3], [4]. How GBS causes meningitis remains incompletely understood [5] and efforts should be made to characterize the underlying pathogenic mechanisms that are essential for crossing the blood – brain barrier (BBB). The functional site of the BBB is the endothelial lining of the brain capillaries constituted by microvascular endothelial cells (BMECs). Blood-borne pathogens may enter the central nervous system (CNS) through multiple mechanisms of transmigration across the brain vasculature such as intercellular (paracellular) and/or transcellular passage, disruption of the endothelial barrier, and leukocyte-facilitated transport by infected phagocytes (also named Trojan horse) [6], [7]. GBS interaction with hBMECs is the primary step in the pathogenesis of meningitis where bacterial transcytosis, endothelial injury, and inflammatory mechanisms may combine to disrupt the BBB [8].

It has been recognized that several human bacterial pathogens gain a survival advantage by interacting with components of the host plasminogen system [9], [10]. Plasminogen is a central component of the fibrinolytic system and is found in plasma and extracellular fluids at concentrations of approximately 2 μM. Upon activation, plasminogen is converted to the serine protease plasmin that is able to degrade fibrin clots, connective tissue, extracellular matrix (ECM), and adhesion proteins. However, several pathogenic microbes manipulate the host plasminogen system to promote their dissemination across several host tissue barriers [9], [10], [11], [12] including the endothelium [13], [14]. Acquisition of a surface-associated plasmin activity by a pathogen likely increases its ability to penetrate the ECM and disseminate to distal sites in the host, a process well documented in the case of S*treptococcus pyogenes* [15], [16], *Borrelia burgdorferi* [17], [18], and *Yersinia pestis* [12], [19]. We have demonstrated that GBS can bind human plasminogen and that this cell-surface associated proenzyme is converted into plasmin by host-derived activators, a process contributing to bacterial virulence [20]. Plasmin is a broad-specific protease that can also activate other proteolytic enzymes such as the matrix metalloprotease that degrades the tight junction components of microvascular endothelial cells [9]. This destruction may favor the passage of bacteria across the vasculature of the CNS and this scenario was proposed as the mechanism of translocation of *B. burgdorferi* through the BBB [14]. We hypothesized that modulation of the host plasminogen system by GBS could play a role in the penetration of the BBB and development of meningitis. In this study, we showed that the presence of plasmin(ogen) on GBS cell surface increases its ability to adhere to, invade, and traverse hBMECs, and enables a high brain penetration in infected neonates. The bacterial invasiveness was reduced following treatment with epsilon-aminocaproic acid (εACA), a lysine analogue that efficiently inhibits the specific binding of plasminogen to cells by competing with lysine-binding-sites [21]. Moreover, addition of εACA to the drinking water of mothers conferred protection to their progenies against GBS infection. This study identifies the interaction of GBS with the host plasminogen system as a key mechanism involved in the BBB transmigration and subsequent CNS invasion. It opens new avenues for the development of innovative strategies to prevent bacterial invasion of the CNS.

## Materials and Methods

### Bacteria strain

Group B Streptococcus (GBS) serotype III virulent strain NEM316 (serotype III, ST-23) was cultured at 37°C in Todd-Hewitt (TH) broth or agar (Disco Laboratories) containing 5 μg/mL of colistin sulphate and 0.5 μg/mL of oxalinic acid (Streptococcus Selective Supplement, Oxoid).

### Mice

Male and female BALB/c mice, purchased from Charles River Laboratories (Barcelona, Spain), were bred at the animal facilities of the ICBAS (Porto, Portugal).

### Ethics statement

This study was carried out in strict accordance with the recommendations of the European Convention for the Protection of Vertebrate Animals used for Experimental and Other Scientific Purposes (ETS 123) and 86/609/EEC Directive and Portuguese rules (DL 129/92). The animal experimental protocol was approved by the competent national authority Direcção Geral de Veterinária (DGV) (Protocol Permit Number: 0420/000/000/2008). All animal experiments were planned in order to minimize mice suffering.

### Culture of human endothelial cells

Human brain microvascular endothelial cell (hBMEC) line was derived from primary cultures of hBMEC transfected with SV40 large T antigen [22] and was cultured as previously described [23]. Cell culture flasks or twenty-four well tissue culture plates were precoated with rat tail collagen (BD Pharmingen) to support the hBMEC monolayers. Viability of hBMEC was assessed by examining cellular morphology and trypan blue exclusion. Cultures were incubated at 37°C in a humid atmosphere of 5% CO2 and split in 1:5 ratio by using trypsin-EDTA (Sigma-Aldrich) when semiconﬂuence was reached. HBMECs were transferred into a collagen-coated 24-well tissue culture plate at a seeding density of 10^5^ cells in growth medium and left for at least 12 hours (h). Prior to each assay, the monolayers were washed three times with Hanks Balanced Salt Solution (HBSS) (Sigma-Aldrich).

### Plasminogen binding and activation on GBS cell surface

Log-phase GBS cells were pelleted, washed three times in sterile PBS, and approximately 2×108 GBS cells were incubated for 1 h at 37°C with 1 μg/mL of purified human plasminogen (Calbiochem) or human plasma. Thereafter, the bacteria were washed with sterile PBS to remove unbound plasminogen and, when incubated with purified plasminogen, tissue plasminogen activator (tPA) (Sigma-Aldrich) was added (20 nM in PBS) for an additional 1 h to convert plasminogen into plasmin. In some experiments, GBS cells were treated for 30 min at 37°C with 200 mM εACA (Sigma-Aldrich) before incubation with plasminogen plus tPA to inhibit GBS cell surface binding. The bacteria were then washed three times in sterile PBS and resuspended at the desired density in serum-deprived fresh cell culture medium without antibiotics.

### Detection of plasminogen bound to GBS cell surface

Plasminogen conjugation with FITC was performed by incubation for 1 h at 30°C in darkness of 400 μg of human plasminogen with 40 μg of FITC (Sigma-Aldrich) in 500 μL of 1 M sodium carbonate buffer, pH 9.2. GBS cells were cultured to log- phase, washed twice in PBS and then fixed in PBS containing 1% paraformaldehyde. After an incubation period of 20 min at 4°C, fixed bacteria were washed twice with PBS and incubated for 30 min at 37°C with different amounts of FITC-conjugated human plasminogen (10, 20 and 50 µg) in 100 µL (final volume). Thereafter, the cells were washed, resuspended in PBS, and analyzed by fluorescence-activated cell sorter (FACS) on an Epics XL cytometer (Beckman Coulter). Data were analyzed using the Expo32 software.

### Bacterial growth curves

Approximately 10^6^ GBS cells, with or without pre-incubation with human plasminogen plus tPA, or pre-incubated with human plasma, were suspended in hBMEC growth medium without antibiotics, and plated on a 24-well tissue culture plate. GBS cells were quantified at different time intervals (15, 30, 45 minutes and every hour up to 6 h) by platting appropriate dilutions of the suspension onto agar plates.

### Bacterial adherence and invasion assays in hBMECs

HBMECs invasion and adherence assays were performed as previously described [24] with the following modifications. HBMECs were infected with either 10^6^ cells of plasmin(ogen)-treated GBS, plasma-treated GBS or untreated GBS in 0.5 ml of serum-deprived fresh cell culture medium without antibiotics to give a multiplicity of infection (MOI) of 10 (10 bacteria per hBMEC). In a separate experiment, hBMECs were incubated in human plasma and infected with 10^6^ cells of untreated GBS. The cultures were maintained at 37°C in a humidified chamber containing 5% CO_2_ and, at indicated time points, were washed three times with HBSS to remove any nonadherent bacteria. For invasion assays, the cultures were further incubated for additional 2 h at 37°C in 5% CO_2_ with 0.5 mL of hBMEC culture medium supplemented with antibiotics (penicillin 5 µg/ml and streptomycin 100 µg/mL) to kill extracellular bacteria. After this incubation period, the monolayers were washed with HBSS, 0.1 mL of trypsin-EDTA solution was added, and the mixture was incubated for 10 min at 37°C. Then, 0.4 mL of 0.025% Triton X-100 was added and each hBMEC monolayer was disrupted by repeated pipetting to release intracellular bacteria. For adhesion assay, total hBMEC-associated (invasive plus surface adherent) GBS were quantified as for the cellular invasion assay but the 2-h incubation with antibiotics was omitted. The number of invasive and adherent bacteria was determined by plating appropriate dilutions of the lysate onto agar plates that were incubated overnight at 37°C. The number of adherent GBS to hBMECs was then determined by subtracting the intracellular bacteria from the total cell-associated (intracellular plus surface-adherent).

### HBMEC injury assay

HBMECs were infected, as in the previous assay, with 10^6^ cells of plasmin(ogen)-treated or untreated GBS in 0.5 ml of serum-deprived fresh cell culture medium without antibiotics to give a MOI of 10. In a separate experiment, hBMECs were incubated in human plasma and infected with 10^6^ cells of untreated GBS. At indicated time-points of infection, the cells were gently washed with pre-warmed PBS. Adherent cells were dyed with Neutral Red medium (40 µg/mL in complete medium) and incubated for further 3 h at 37°C in a humidified chamber containing 5% CO2. The medium was then carefully removed and the cells were washed twice with pre-warmed PBS. The dye in each well was extracted with de-staining solution (1% acetic acid/50% ethanol) and the cells were allowed to stand for 10 min at room temperature. The absorbance was read with a spectrophotometer at a wavelength of 540 nm. The cell injury was evaluated by the percentage of viability observed in infected cells relative to uninfected cells. In some experiments, the hBMEC injury was confirmed by measuring the release of lactate dehydrogenase (LDH) into the supernatant of the hBMEC infected cells. In this case, hBMECs were incubated with plasmin(ogen)-treated and untreated GBS for 120 min, and the culture supernatant was collected and centrifuged for 10 min at 12,000 g to remove the cells. LDH activity was determined using a commercial kit (Sigma-Aldrich) according to the manufacturer's guidelines.

### Migration assay of GBS across hBMEC

A endothelial BBB model *in vitro* was established by cultivating the hBMECs on collagen-coated polycarbonate transwell membrane inserts with a pore size of 3 µm (Corning). This *in vitro* model of the BBB allows separate access to the upper chamber (blood side) and lower chamber (brain side) and mimics GBS penetration into the brain. The hBMEC monolayer was grown by seeding 500 µL of growth medium containing 5×10^5^ cells in the upper channel and 1,500 µL growth medium in the bottom chamber of 12-well tissue culture inserts. The hBMEC were grown to confluence for at least 5 days at 37°C in a humidified chamber containing 5% CO_2_. At this point, the transendothelial electric resistance (TEER) of this monolayer was around 200–250 Ω cm^−2^, as measured with a Millicell ERS-2 (Electrical Resistance System) meter (Millipore). In our experiment, only monolayers with TEER greater than 200 Ω cm^−2^ were used. Prior to the assay, hBMECs were washed and serum-free culture medium without antibiotics was added. Log-phase GBS cells (10^6^ CFU) untreated or treated with human plasmin(ogen) were applied to the apical chamber (total volume of 500 µL) and the monolayers were incubated at 37°C in a humidified chamber containing 5% CO_2_. At 1 and 2 h post-infection, the lower chamber medium was entirely removed and plated onto TH agar to enumerate the number of bacteria crossing the hBMEC monolayer. Simultaneously, the integrity of the hBMEC monolayer was assessed by TEER measurement. Three measurements were made at each time-point for every sample.

### Determination of GBS cell surface plasmin-like activity

HBMECs cultured in 24-well plates were infected with 10^6^ cells of plasmin(ogen)-treated or untreated GBS resuspended in growth medium, or with 10^6^ cells of untreated GBS in whole human plasma. In some experiments, GBS cells were incubated for 1 h at 37°C with 200 mM εACA before plasminogen and tPA treatment. The cultures were incubated at 37°C in a humidified chamber containing 5% CO_2_. At the desired time point post-infection, the supernatant was removed and centrifuged for 10 min at 12,000 g to recover GBS cells. The bacterial cells were then washed once in PBS and resuspended in the chromogenic plasmin substrate Val-Leu-Lys-p-nitroanilide (400 µM final concentration) (S2251, Chromogenix). Following a 24 h incubation period at 37°C, the cells were pelleted and the optical density of the supernatant was read at 405 nm using a microplate reader. All samples were loaded into triplicate wells. At each time-point, the bacterial growth in the supernatant was also quantified by plating appropriate dilutions of the supernatant onto TH agar plates.

### Challenging infections of newborn mice

2-days old BALB/c mice were infected intraperitoneally (i.p.) with 5×10^6^ cells of plasminogen-treated and untreated GBS in a maximum volume of 40 μL. The brain of infected pups, aseptically removed at indicated time points, was homogenized in PBS and serial dilutions were plated on TH agar to enumerate bacterial Colony-forming units (CFU). To inhibit plasminogen binding to GBS, εACA was added (12 g/L) to the drinking water of pregnant female BALB/c mice from the gestational day 15 to the end of the experiment (sacrifice of the pups). As control experiment, normal water was given to pregnant female. The neonates were kept with their mothers throughout the end of the experiments. Females were monitored closely during gestation and the day of delivery was recorded. Two days after the birth, the pups were infected with 5×10^6^ cells of untreated GBS and the numbers of CFU in blood, liver, lung, and brain were determined 18 h post-infection. The organs aseptically removed at indicated time points were homogenized in PBS and serial dilutions were plated on TH agar to enumerate bacterial CFU. Survival curves were determined in a 7-day experiment period.

### Statistical analysis

All graphs were generated using GraphPad Prism software (GraphPad Software). Means and standard errors of the means (SEM) were calculated. Student's t test was used to analyze the differences between groups. Survival studies were analyzed with the log-rank test. Both tests used GraphPad software. A P value of <0.05 was considered statistically significant.

## Results

### Role of plasminogen system in adhesion and invasion of the human brain endothelial cells by GBS

**Figure 1:**
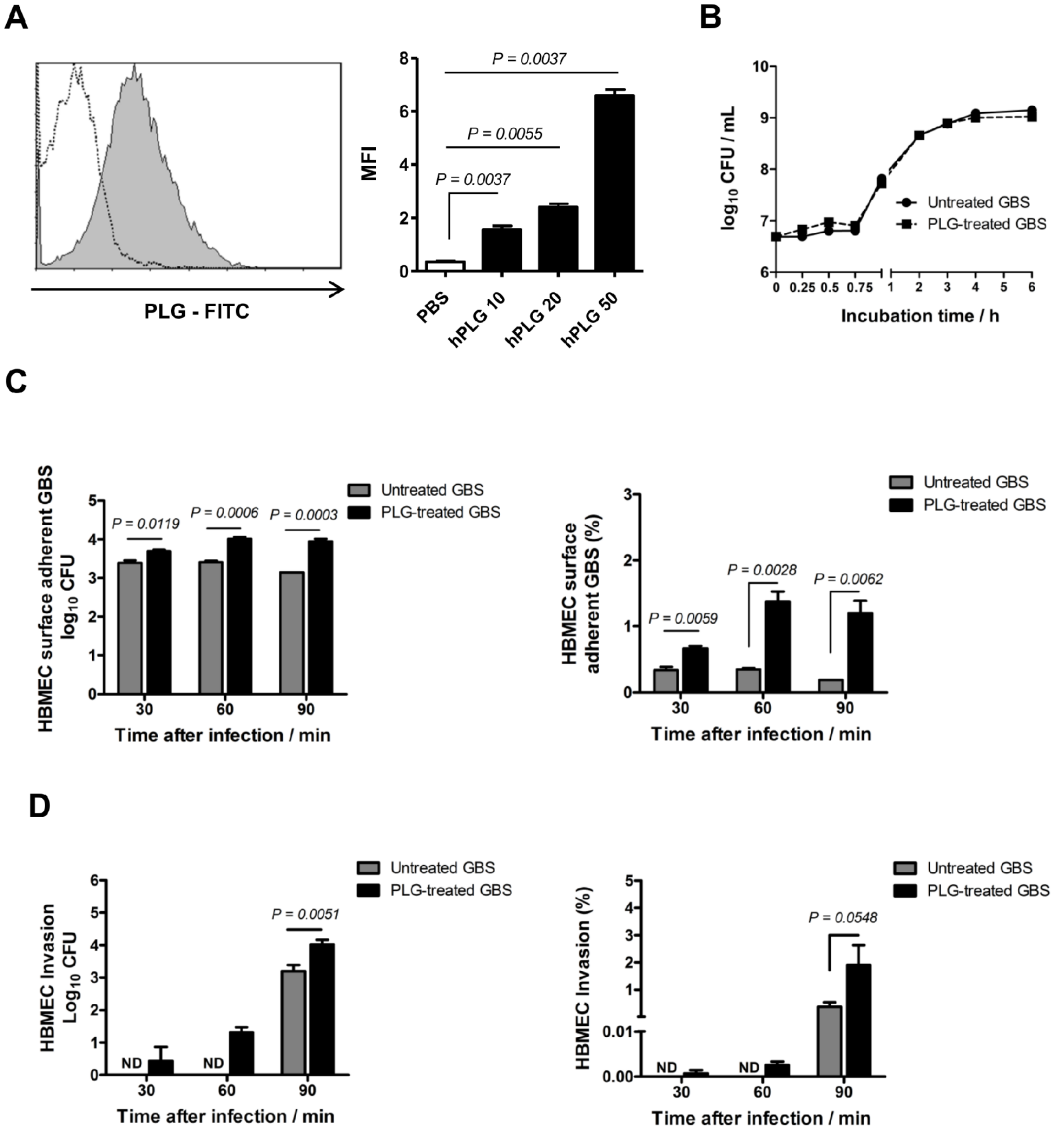
Plasmin(ogen)-coated GBS displays enhanced abilities to adhere to and invade hBMECs *in vitro*. (A) GBS cells were incubated with FITC-labeled human plasminogen (hPLG) (grey filled histogram) or PBS (white dotted histogram). Plasminogen binding was measured by a FACScan cytometer as the increase in FITC mean ﬂuorescent intensity (MFI). Each histogram shows cell number as a function of relative fluorescence obtained for 10,000 events per population. Results are shown for 10, 20, and 50 µg of FITC-conjugated hPLG. (B) Representative growth curves of GBS preincubated without (untreated GBS) or with (PLG-treated GBS) plasminogen plus tPA in complete hBMEC growth medium. Data are from a experiment performed in triplicate that is representative of three independent experiments. Each point is the mean of three samples ± SEM. (C and D) HBMEC monolayers were infected with 10^6^ cells of GBS preincubated without (untreated GBS) or with (PLG-treated GBS) plasminogen plus tPA (MOI of 10 bacteria per cell). (C) HBMECs surface adherent GBS cells and (D) intracellular bacteria were isolated and enumerated after 30, 60, and 90 min of infection. The percentages of hBMECs surface adherent GBS and intracellular bacteria are expressed relative to the initial inoculums. Data are the mean + SEM of three independent experiments. Statistical differences (P values) are indicated; ND – not detected.

GBS adherence to BMECs constitutes the initial step in the invasion into the CNS [24] and increased invasiveness is observed when plasmin(ogen) is bound to GBS surface [20]. Therefore, we set out to determine whether the plasminogen system plays a role in the invasion of CNS by GBS. For that purpose, an hBMEC line that maintains the morphological and functional properties of primary brain endothelium [25], [26] was used to evaluate the adhesion, invasion and injury of hBMECs after infection of GBS preincubated with or without human plasminogen and tPA. We first demonstrated that human plasminogen efficiently binds to GBS cell surface (Figure 1A) and that the growth rates of the plasmin(ogen)-treated and untreated GBS were identical (Figure 1B). We next performed the adhesion and invasion assays in hBMECs by plasmin(ogen)-treated or untreated GBS. At all time points tested, a significant increase in the percentage of bacterial cells adhered to endothelial cells was observed with plasmin(ogen)-treated GBS, compared with untreated bacterium (Figure 1C). Moreover, at 30 and 60 min post-infection, invasion of hBMECs was only observed with plasmin(ogen)-treated bacteria (Figure 1D) and, 90 min post-infection, the percent invasion was significantly higher with plasmin(ogen)-treated GBS compared to untreated bacteria. These results show that plasmin(ogen) bound to GBS surface increase the ability of the bacterium to adhere to and invade hBMECs.

### Plasmin(ogen) bound to GBS surface induces hBMEC injury

**Figure 2:**
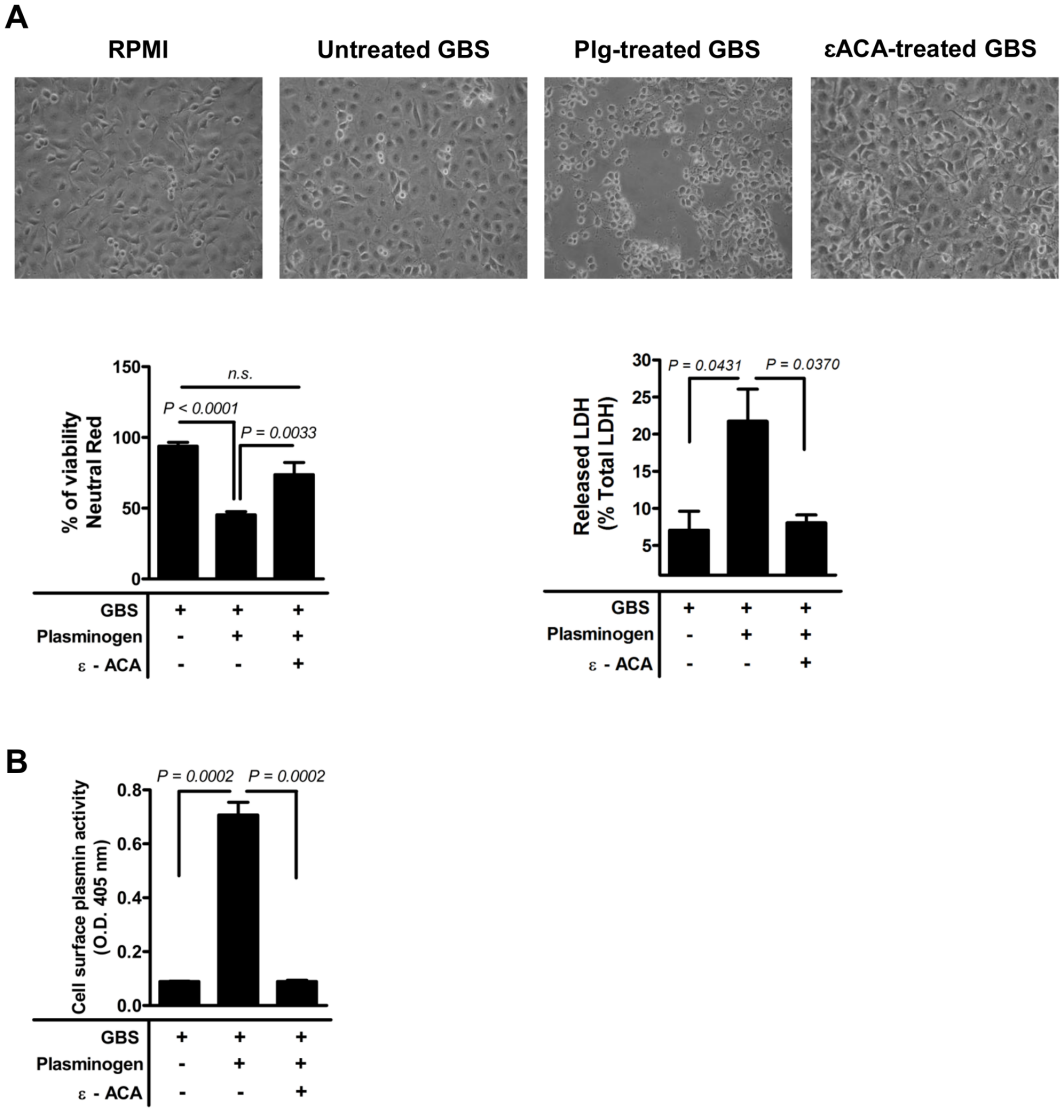
Plasmin(ogen)-coated GBS induces hBMECs detachment and injury. GBS cells were preincubated without (untreated GBS) or with (PLG-treated GBS) plasminogen plus tPA, or pre-treated with εACA prior to plasminogen plus tPA incubation (εACA-treated GBS). HBMECs monolayers were infected with 10^6^ CFU (MOI of 10) of untreated GBS, PLG-treated GBS or εACA-treated GBS, for 120 min at 37°C; uninfected cells were used as negative controls. (A) *Upper panel*: representative microscopic photos of the average cell density were taken at ×100 magnification for visualization purposes. *Lower left panel*: percentage of viable cells, determined by the neutral red assay, expressed relative to the number of viable cells observed in uninfected control. *Lower right panel*: Cell viability determined by measuring the LDH release. Data represents mean the values normalized to the mean 100%-death control + SEM from an experiment performed in triplicate that is representative of three independent experiments. (B) Plasmin-like activity in bacterial cell surface. The plasmin activity in GBS surface was assessed following incubation with its specific chromogenic substrate S-2251 and determination of the absorbance at 405 nm. Data represents mean + SEM from an experiment performed in triplicate that is representative of three independent experiments.

We have previously shown that plasminogen recruited to the GBS cell surface by plasminogen receptors is converted into plasmin by host-derived activators, thus generating a proteolytic bacterium [20]. Therefore, we next questioned whether GBS surface-bound plasmin(ogen) induced the hBMECs monolayer disruption. The cell viability was assessed after infection of GBS preincubated with or without plasminogen plus tPA. The quantification of hBMECs viability, assessed 120 min post-infection by the neutral red uptake assay, showed that the percentage of viable hBMECs infected with untreated GBS was greater than 95% (Figure 2A, *lower left panel*). In contrast, a noticeable increase in hBMECs detachment was observed at 120 min post-infection with plasminogen-treated GBS that was associated with a 50% decrease in hBMECs viability (*P<*0.0001). However, when GBS cells were incubated with εACA before plasminogen and tPA treatments only 20% of decrease in hBMECs viability was observed (Figure 2A, *lower left panel*). Moreover, the release of LDH by hBMECs was significantly higher with plasmin(ogen)-treated GBS compared with untreated bacteria (Figure 2A, *lower right panel*). Cell surface-bound plasmin is exploited by bacteria for proteolytic degradation of components of the ECM, basal membrane, and host tissues [27]. We therefore determined the plasmin-like activity of GBS cell surface when hBMECs were infected with plasmin(ogen)-treated or untreated GBS. As shown in Figure 2B, the bacteria coated with plasmin(ogen) displayed a significantly greater proteolytic activity at the time point tested, which is abrogated when the GBS cells were incubated with εACA before plasminogen and tPA treatments. These results indicate that the cell surface-bound plasmin(ogen) endows the bacteria with proteolytic activity that promotes disruption of hBMECs monolayer.

### Human plasma incubation increases the ability of GBS to invade and degrade hBMECs

**Figure 3:**
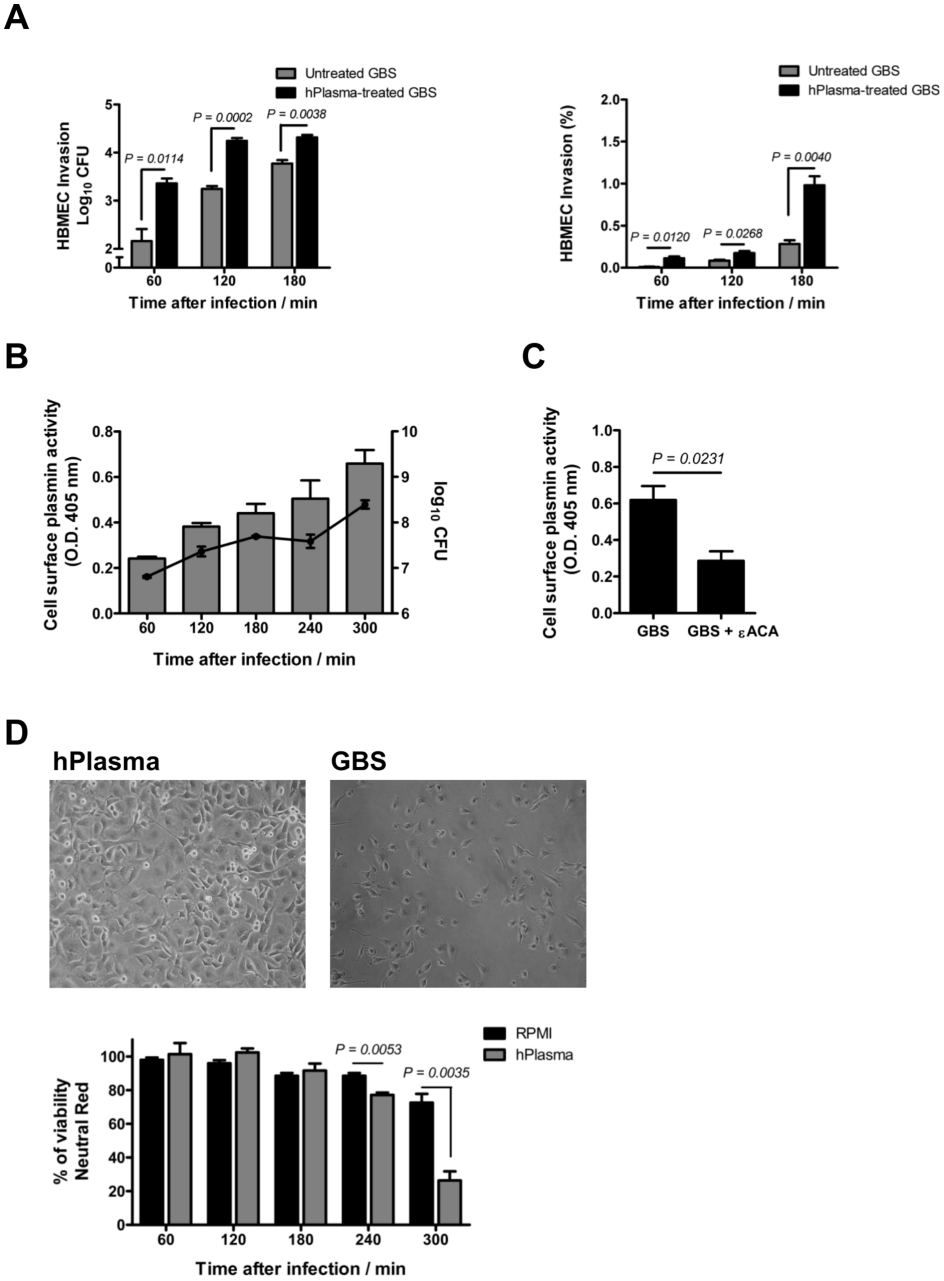
Incubation with human plasma increases the ability of GBS to invade and degrade hBMECs. (A) HBMECs monolayers were infected with 10^6^ GBS CFU (MOI of 10) pre-incubated or not with whole human plasma and bacterial invasion was determined at the indicated time points and expressed in log 10 CFU/mL (left) or in percentage of intracellular bacteria relative to the initial inoculum. (B) HBMEC monolayer were infected with 10^6^ GBS CFU (MOI of 10) in whole human plasma and the plasmin-like activity of GBS cells was assessed as described in Figure 2C (bars are the mean values of plasmin activity + SEM) and the bacterial CFU were determined at the same time points (line represents the mean numbers of bacterial CFU ± SEM). (C) HBMECs monolayers were infected with 10^6^ GBS CFU (MOI of 10) preincubated (GBS + εACA) or not (GBS) with 200 mM εACA in whole human plasma for a 300 min period at 37°C. The acquisition of cell surface plasmin activity was detected as described in Figure 2C and results are the mean values + SEM of the plasmin activity determined in one experiment performed in triplicate. These data are representative of three independent experiments. Statistical differences (P values) are indicated. (D) *Upper panel*: representative microscopic photos of the average cell density after 300 min of infection (for visualization purposes, magnification was at 100X). *Bottom panel:* the percentage of viable cells, assessed by the neutral red assay, was determined as described in Figure 2A. Data are the mean + SEM and are representative of three independent experiments. Statistical differences (P values) are indicated.

Numerous unrelated streptococcal surface proteins are involved in the binding of plasminogen including the glycolytic enzymes enolase, phosphoglycerate kinase, glyceraldehyde-3-phosphate dehydrogenase (GAPDH), and phosphoglycerate mutase [28]. We have previously shown that plasma plasminogen is efficiently recruited at the GBS cell surface [25] and, more recently, have identified several plasminogen-binding receptors on the GBS surface among which enolase and GAPDH were predominant in binding plasminogen (Magalhães *et al.*, unpublished data). To provide a more physiologically relevant experimental set-up, the invasion and hBMECs viability assays were studied following incubation of the hBMECs monolayer in whole human plasma that contains plasminogen at a physiological concentration (2 μM) [29]. GBS invasion of the hBMECs was significantly greater at all time points analyzed when the bacteria were pretreated with human plasma compared to untreated cells (Figure 3A). This difference was not due to increased bacterial growth since untreated GBS or human plasma-treated GBS displayed similar growth rates (data not shown). We next determined whether the interaction of GBS with human plasma in the presence of hBMECs can lead to acquisition of cell surface plasmin activity. The proteolytic activity on the GBS cell surface was assessed using the chromogenic substrate S2251 at different time points (60, 120, 180, 240, and 300 min) of co-culture. As shown in Figure 3B, GBS co-cultured with hBMECs in human plasma acquired an increasing surface plasmin-like activity over the incubation period that reflects bacterial growth. To ensure that the observed GBS cell surface plasmin activity was due to binding and activation of the plasminogen present in human plasma, we determined at 300 min post-infection the proteolytic activity of GBS cells previously incubated with εACA prior to hBMECs infection. As shown in Figure 3C, pre-incubation of GBS with εACA significantly (*P = 0.0231*) inhibited the acquisition of cell surface plasmin activity by GBS. The inhibition was not complete, presumably because plasmin(ogen)-coated GBS can arise after bacterial multiplication in the plasma. No difference between the bacterial CFU at this time point was observed between εACA-treated and untreated GBS indicating that the εACA treatment did not modify the bacterial growth (data not shown). Moreover, as shown in Figure 3D, infection of hBMECs with GBS in human plasma leads to a significant cell detachment, as observed at 240 and 300 min post-infection. These experiments showed that plasminogen present in plasma is recruited to the bacterial cell surface and is converted into plasmin by host-derived activators leading to hBMECs injury.

### GBS-surface bound plasmin(ogen) promotes transmigration across hBMECs

**Figure 4:**
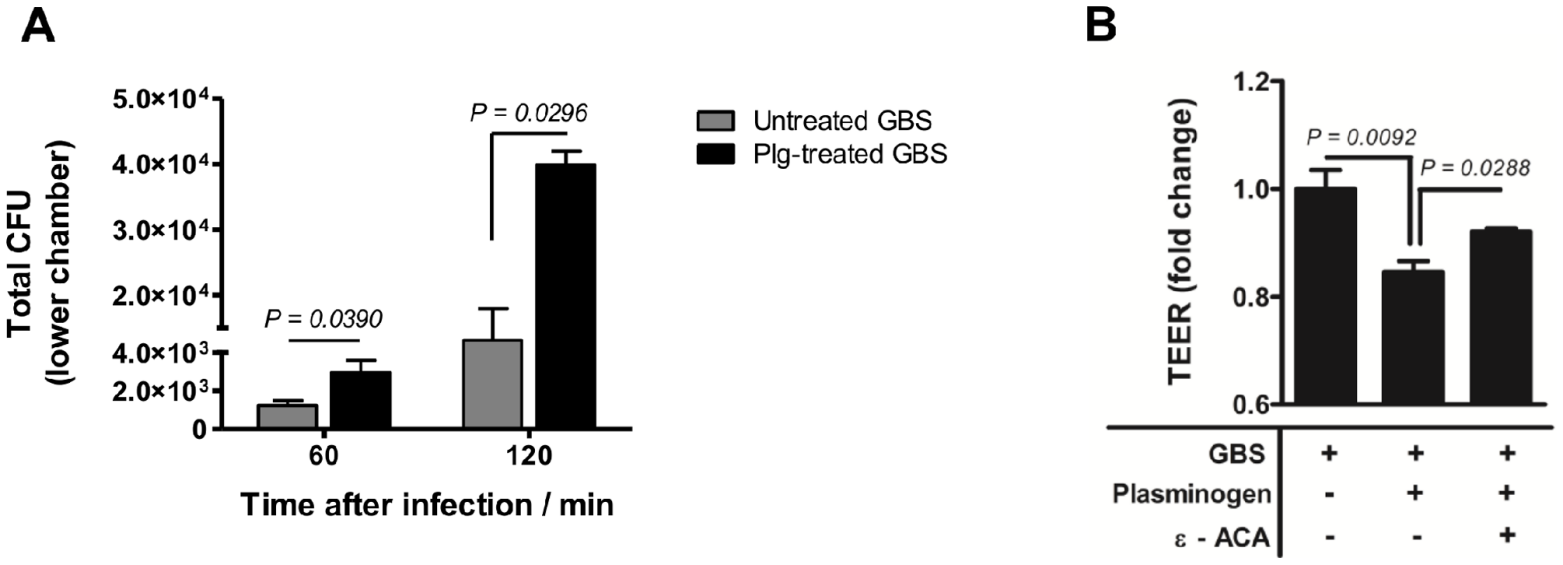
Transmigration of GBS across hBMECs. (A) Confluent hBMECs monolayers grown in the upper chamber of Transwell inserts were infected for a 2 h period with 10^6^ GBS cells preincubated without (untreated GBS) or with (PLG-treated GBS) plasminogen plus tPA. The total lower chamber medium was collected at the indicated time points and total GBS CFU were enumerated. (B) The integrity of the HBMEC monolayers infected with 10^6^ GBS CFU previously incubated with plasminogen plus tPA (PLG-treated GBS), untreated (untreated GBS) or pre-treated with εACA prior to plasminogen plus tPA incubation (εACA-treated GBS) was monitored by measuring the change in TEER. Data are the mean values + SEM of at least two experiments. Statistical differences (P values) are indicated.

It was reported that GBS adheres to and invades hBMECs [24], but the factors that contribute to the penetration of the BBB remain to be characterized. To investigate the role of plasminogen system in this process, an *in vitro* assay mimicking GBS migration across the BBB was performed in a transwell system. As shown in Figure 4, a time-dependent increase in GBS crossing through the hBMECs monolayer was observed both with untreated and plasmin(ogen)-treated bacteria. However, plasmin(ogen) captured by the GBS cell surface significantly increased the ability of this bacterium to traverse across the hBMECs monolayer. After 60 and 120 min of infection, plasmin(ogen)-treated bacteria displayed a 3- and 2-fold increase in their ability to migrate through the hBMECs monolayer, respectively, as compared to untreated GBS cells (Figure 4A). Consistently, a significant reduction of the trans-epithelial electric resistance (TEER) was observed when GBS cells were incubated with plasmin(ogen), as compared to untreated bacteria, and this effect was abolished by εACA (Fig. 4B). These results suggest that the plasminogen system contributes to GBS migration across the hBMEC monolayer by disrupting the cellular integrity.

### Role of the host plasminogen system in BBB penetration in a murine model of infection

**Figure 5:**
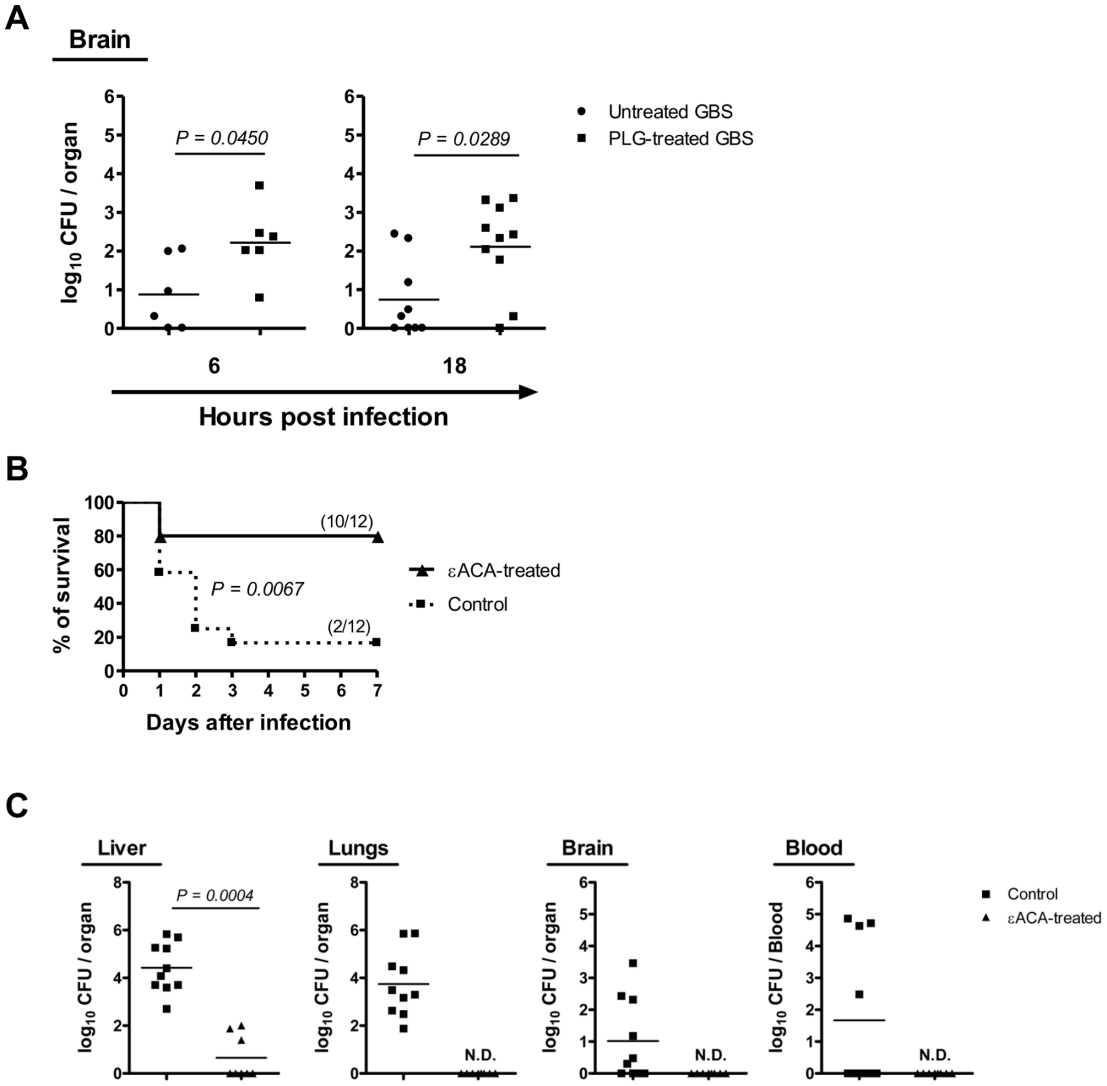
Plasmin(ogen)-coated GBS displays enhanced abilities to invade the central nervous system. (A) Neonatal BALB/c mice were infected i.p. at 48 h after birth with 5×10^6^ CFU of GBS incubated with (PLG-treated GBS) or without (untreated GBS) plasminogen plus tPA. GBS CFU were determined in the brain of neonates at 6 and 18 h post-infection. Results from individual mice are shown. Statistical differences (P values) between groups are indicated. (B and C) Pregnant BALB/c mice, from the gestational day 15 until the end of the experiment, were given drinking water containing εACA (12 g/L) or normal water (control group). The newborns were kept with their mothers throughout the experiments. Two days after the birth, the pups were infected with 5×10^6^ cells of untreated GBS. (B) Kaplan-Meier survival curves of neonatal mice born from εACA-treated or control mothers. The numbers between parentheses represent the number of animals that survive versus the total number of infected animals. Results represent data pooled from two independent experiments. (C) GBS CFU recovered at 18 h post-infection in the liver, lungs, blood and brain of pups. Results from individual mice are shown. Statistical differences (P values) between groups are indicated. ND – not detected.

The *in vitro* results described above support our hypothesis that interaction of GBS with the host plasminogen system enhances the ability of this bacterium to invade the CNS *in vivo*. To confirm this hypothesis, neonatal mice were infected by intraperitoneal route (i.p.), 48 h after birth, with 5×10^6^ cells of plasmin(ogen)-treated or untreated GBS. The bacterial load was determined at 6 h and 18 h post-infection in the brains of the neonatal mice from both groups. As shown in Figure 5A, 6 and 18 h after infection, the brains of the neonates mice challenged with plasmin(ogen)-treated GBS displayed higher bacterial counts than those challenged with untreated GBS (*P* = *0.0450 and P = 0.0289,* respectively). No difference in bacterial counts was observed in the blood of pups infected with untreated or plasmin(ogen)-treated GBS, excluding the possibility that increased brain penetration was due to increased levels of bacteremia (data not shown).

We further tested whether the increased entry of plasmin(ogen)-treated GBS into the brain of infected pups was associated with cell-surface plasmin(ogen) binding. Based on the knowledge that orally administered εACA is almost entirely absorbed from the gastrointestinal tract and is rapidly detected in plasma [30], we developed an *in vivo* assay in which plasminogen binding to GBS was blocked by adding εACA in the drinking water of pregnant mice, while the control group was given normal water. Pups born from εACA – treated or control females were infected i.p., 48 h after birth with 5×10^6^ untreated-GBS cells. The pups were maintained with their mothers throughout the experiments. Ten out of the 12 mice born from εACA – treated mothers survived the infection (83.3% survival) whereas all but two infected pups succumbed to GBS challenge in the control group (16.7% survival) (*P = 0.0067*) (Figure 5B). Moreover, as depicted in Figure 5C, a significantly lower CFU of GBS were recovered 18 h after infection from the liver of pups born from εACA–treated mothers, as compared to the control group. Importantly, no bacterial counts were detected 18 h after GBS infection in the lung and brain of pups born from εACA – treated mothers (Figure 5C). Moreover, in pups born from εACA-treated mothers, no CFU were detected in all blood samples, while the blood samples of some control pups were found to have CFU.

Altogether, our results indicate that interaction of GBS with the host plasminogen system contributes to successful crossing of the BBB and penetration into the CNS.

## Discussion

A major limitation to advances in the prevention and treatment of CNS infection is our incomplete understanding of the pathogenesis of this disease and the associated BBB dysfunction. The development of *in vitro* models of BBB crossing and *in vivo* animal models of experimental haematogenous meningitis have shed some light on the mechanisms of microbial traversal of the BBB, the key step leading to CNS infections. Concerning the pathophysiology of GBS, the precise mechanisms whereby this bacterium leaves the bloodstream and access to the CNS remain incompletely understood. A number of GBS surface proteins including pili [31], the fibrinogen adhesin FbsA [32], the serine-rich repeat glycoprotein Srr1 [33], the hypervirulent GBS adhesin HvgA [6], [34], the laminin-binding protein Lmb [35], and the lipoteichoic acid anchoring enzyme LagA [36], have been shown to promote *in vitro* adhesion to or invasion of BMECs. A recent study identified the surface glycosaminoglycans as host receptor molecules that interact with the GBS alpha C protein to facilitate the bacterial entry into CNS [37].

We previously reported that GBS can specifically bind human plasminogen that can be subsequently activated to plasmin by host activators, such as uPA and tPA, and generate a proteolytic bacterium that is more virulent [20]. As reported in other bacteria [38], [39] including streptococci [28] we have identified the GBS enolase and GAPDH as predominant cell surface plasminogen binding proteins (Magalhães et al., unpublished data). As these proteins are essential for bacterial growth, the corresponding genes could not be deleted. The ability of invasive pathogens to recruit plasmin(ogen) on their surface constitutes a well described strategy for translocation through tissue barriers and dissemination [9], [10], [12], [40]. In the present study, we demonstrated that GBS utilizes the host plasminogen system to promote bacterial migration across BBB and entry into the CNS. We showed that, early after infection, GBS surface-bound plasmin(ogen) increased the bacterial adherence to and invasion of hBMECs monolayer and subsequently induced hBMECs injury and disruption by endowing the bacteria with host-derived proteolytic activity. Plasmin bound to GBS cell surface is enzymatically active, as measured by the degradation of chromogenic specific plasmin substrate. Several studies have shown that the acquired and surface-bound proteolytic activity endows the bacterium with the capacity to degrade components of the ECM [41] and penetrate endothelial monolayers, including the BBB [13], [14], [42], [43], [44]. Accordingly, plasmin-mediated penetration of tissue barriers leading to brain invasion was reported in the case of *B. burgdorferi* [42]. Another important factor for the development of meningitis is the ability of pathogens to cross the BBB as live organisms. Transmission electron microscopy studies with extracellular pathogens (e.g., *Escherichia coli* and GBS) revealed that they transmigrate across hBMECs monolayer in membrane-bound enclosed vacuoles [24], [45]. Our results suggest that these two potential mechanisms can function sequentially. The increased internalization detected early during the infection process should favor the bacterial transcytosis, as reported by others [22], [46], and the cell injury and detachment of hBMECs observed later should favor bacterial transmigration between injured cells. The loss of BBB integrity had also been reported with the toxicity of bacterial products and/or the activation of host inflammatory mediators [47], [48]. Here, we provide evidences that hijacking of the host plasminogen system to generate a proteolytic bacterium constitutes another mechanism enabling endothelial cell injury. Indeed, we showed that incubation of hBMECs and GBS in human plasma resulted in the acquisition of a plasmin activity at the bacterial surface. This surface modification should facilitate the GBS traversal of the extracellular matrix barriers and the tissue penetration and, consequently, led to increased bacterial invasiveness. Our *in vitro* results were confirmed *in vivo* in a neonatal murine model of GBS infection where an increased CNS dissemination was observed in neonates infected with GBS cells exhibiting surface-bound plasmin(ogen). This indicates that recruitment of the host plasminogen to the bacterial surface generates a proteolytic bacterium that, after conversion to plasmin, possesses an increased ability to traverse the BBB. Since εACA has been used as an anti-fibrinolytic agent in humans, this molecule was added to the drinking water of mother's mice. The liver of GBS-infected pups born from εACA-treated mothers was much less colonized than those born from untreated progenitors. Remarkably, no bacterium was recovered from the lungs and the brains of pups born from εACA-treated mothers, whereas those of pups from untreated mothers were found to possess detectable CFUs. Moreover, pups born from εACA-treated mothers were free of detectable bacteremia which could indicate that GBS-associated plasmin(ogen) facilitate bacterial access to the vasculature with systemic spread, as described for Group A Streptococcus [16]. Remarkably, the treatment of mothers with εACA improved neonatal survival from 16.7 to 83.3%. Our results combined with previous reports suggest that the ability to recruit the host plasmin(ogen) could constitute a strategy utilized by unrelated meningeal pathogens such as GBS (this work), *Streptococcus pneumoniae* [49], and *B. burgdorferi* [13], [14].

Overall, our findings suggest that plasmin(ogen) bound at GBS surface facilitates bacterial penetration of CNS. To our knowledge, this is the first report that identifies the interaction of GBS with the host plasminogen system as one of the key events in the pathogenesis of CNS infections. Moreover, our results suggest that therapies aimed at neutralizing the activation of plasminogen system at GBS surface could be beneficial in preventing development of meningitis.
